# MRI-based Quantitative Collateral Assessment in Acute Stroke: A Comparison with Single-phase CTA in Drip-and-ship Patients with Serial Imaging

**DOI:** 10.1007/s00062-024-01456-6

**Published:** 2024-09-24

**Authors:** Christoph Polkowski, Niklas Helwig, Marlies Wagner, Alexander Seiler

**Affiliations:** 1 Institute of Neuroradiology, Goethe University Frankfurt, Frankfurt, Germany; 2 Department of Neurology, Goethe University Frankfurt, Frankfurt, Germany; 3 Department of Neurology, University Hospital Schleswig-Holstein, Campus Kiel, Kiel, Germany; 4 Department of Neurology and Neurovascular Center, University Hospital Schleswig-Holstein, Campus Kiel, Arnold-Heller-Straße 3, 24105 Kiel, Germany

**Keywords:** Large-vessel occlusion, Collaterals, Perfusion-weighted imaging, Computed tomography angiography, Functional outcome

## Abstract

**Purpose:**

In acute ischemic stroke with large-vessel occlusion (LVO), collateral assessment with single-phase computed tomography angiography (CTA) might underestimate pial collateral supply in a considerable proportion of patients. We aimed to compare time-resolved magnetic resonance imaging (MRI)-based quantitative collateral mapping to conventional collateral imaging with CTA.

**Methods:**

This retrospective single-center study covering a period of 6 years (2012–2018) included drip-and-ship LVO patients who underwent MR imaging after initial imaging evaluation with CT. For MRI-based collateral assessment, T2*-weighted time series from perfusion-weighted imaging (PWI) were processed to compute a quantitative collateral vessel index (CVI_PWI_) based on the magnitude of signal variance across the entire acquisition time. CTA-based collateral scores (Tan and Maas) and CVI_PWI_ were investigated in terms of inter-modality associations between collateral measures, as well as their relationships with stroke severity, infarct volume and early functional outcome.

**Results:**

The final analysis included *n* = 56 patients (*n* = 31 female, mean age 69.9 ± 14.21 years). No significant relationship was found between MR-based quantitative collateral supply (CVI_PWI_) and CT-based collateral scores (r = −0.00057, *p* = 0.502 and r = −0.124, *p* = 0.797). In contrast to CVI_PWI_, CTA-based collateral scores showed no significant relationship with clinical stroke severity and infarct volume. While MR-based CVI_PWI_ was independently associated with favorable early functional outcome in multivariate analysis (OR 1.075, 95% CI 1.001–1.153, *p* = 0.046), CTA-based collateral scores were not significantly associated with outcome.

**Conclusions:**

Since collateral scores based on single-phase CTA do not accurately reflect infarct progression and might underestimate pial collateralization in a relevant proportion of patients, they are not associated with early functional outcome in LVO patients. In contrast, CVI_PWI_ represents a robust imaging parameter of collateral supply and is independently associated with functional outcome.

## Introduction

In acute ischemic stroke (AIS) due to large-vessel occlusion (LVO) of the anterior circulation, pial collateral vessels are the key protective element, which limit infarct progression over time and therefore control the pace of the ischemic tissue damage [1–4]. Since the magnitude of collateral supply is closely related to the effect size of endovascular reperfusion and is therefore strongly predictive of functional outcome [5], exact knowledge about the quality of collateral flow in individual cases may be of major importance for clinical prognostication. This may be especially valid for drip-and-ship patients, which are transferred — sometimes over long distances — to larger stroke centers for endovascular treatment after initial clinical and imaging assessment at a primary care center.

However, current approaches for collateral assessment usually based on single-phase computed tomography angiography (CTA) rely on rather approximative grading scales, which are observer-dependent and do not produce direct quantitative measures of the collateral status [6]. Furthermore, single-phase CTA as used in standard clinical care largely depicts retrograde perfusion of larger arterial branches early after injection of the contrast bolus without detecting later, but still sufficient filling of smaller pial collaterals and cortical anastomoses [7]. These shortcomings of CTA-based collateral assessment might explain why previous research failed to demonstrate a significant relationship between conventional collateral scores and functional outcome in patients treated with EVT for LVO in the advanced time window, although an impact of collateral supply on final infarct volume could be observed [7, 8]. In contrast, quantitative assessment of collateral supply based on perfusion magnetic resonance imaging (MRI) provides a time-resolved and rater-independent parameter of leptomeningeal collateralization [9, 10]. In this study, we aimed to directly compare standard collateral grading based on single-phase CTA with perfusion MRI-based quantitative collateral assessment in drip-and-ship patients with LVO, who underwent serial imaging before endovascular stroke treatment. The main objectives of this study were to evaluate inter-modality statistical associations of collateral measures as well as their associations with imaging-based and clinical outcome parameters including infarct volume and progression, clinical stroke severity and early functional outcome.

## Material and Methods

### Patients

Data from drip-and-ship patients referred to our academic stroke center after detection of LVO on baseline imaging in primary care hospitals between January 2012 and December 2018 were analyzed. The drip-and-ship patients included in this study represent a subgroup of all patients identified in our institutional database, who were admitted in the period 2012–2018 with LVO-AIS and received MR imaging with acceptable image quality for automated postprocessing (*n* = 129). All included patients underwent a standardized stroke MRI protocol based on a former institutional paradigm, according to which MRI was performed for therapeutic decision-making for thrombectomy in cases with longer time intervals (≥ 120 min) between initial imaging and arrival at the tertiary stroke center, older age or severe clinical deficit at presentation. All patients had received CT imaging with CTA for detection of LVO. The study was approved by the local institutional review board (Goethe University Frankfurt, Faculty of Medicine, approval number: 400/18). Informed consent from individual patients was waived because of the retrospective character of the study.

### Image Evaluation for Grading of Collateral Supply and Infarct Volume

Visual collateral assessment on baseline CTA as well as determination of the Alberta Stroke Program Early CT Score (ASPECTS) on non-contrast CT and DWI were performed by two experienced neurological/neuroradiological raters (both with > 12 years of experience), who were blinded to all other patient information. The established scales by Tan [11] and Maas [12] were used for collateral grading. All disagreement on collateral rating was finally resolved by consensus. Besides the ordinal scales with the full range of collateral grades, both scores were dichotomized [7, 13] and used as binary variables for stratification of the patients into subjects with favorable and subjects with poor collateral supply.

### MR Imaging Protocol

Besides diffusion- and perfusion-weighted imaging (DWI and PWI), which are the relevant imaging sequences for this study, the institutional stroke protocol included sequences for anatomical parenchymal and low flow imaging (conventional T2-weighted, fluid-attenuated inversion recovery) plus sequences to detect intracerebral hemorrhage and assess thrombus length (T2*-weighted or susceptibility-weighted imaging). Finally, for the detection of LVO, a 3-dimensional time-of-flight magnetic resonance angiography was included.

DWI data were acquired with a single-shot spin-echo echo-planar imaging (EPI) sequence, using the following parameters: echo time TE = 88 ms, repetition time TR = 4900 ms, flip angle 90°, field-of-view 220 × 220 mm^2^, matrix size 130 × 130, 25 axial slices, slice thickness 5 mm, inter-slice gap 0.5 mm and bandwidth BW = 1425 Hz/pixel. Diffusion sensitizing gradients were applied sequentially with b = 0, b = 500 s/mm^2^ and b = 1000 s/mm^2^. Apparent diffusion coefficient (ADC) maps were calculated using the commercially available scanner software. Perfusion-weighted images were acquired with a T2*-weighted dynamic susceptibility contrast (DSC) gradient-echo EPI sequence. Imaging parameters were TE = 30 ms, TR = 1500 ms, flip angle FA = 90°, field-of-view 230 × 230 mm^2^, matrix size 128 × 128, 19 axial slices, slice thickness 4 mm, 1.2 mm inter-slice gap, BW = 1447 Hz/pixel, acquisition time 1:41 min. The intravenous contrast agent gadobutrol (0.1 mmol/kg; Gadovist® Bayer) was automatically applied by a power injector at a flow rate of 5 mL/s, followed by a bolus (20 mL) of 0.9% saline.

### Image Postprocessing and Analysis for Ischemic Core Volumetry and MR-based Quantitative Assessment of Collateral Supply

Further image postprocessing and analysis were performed automatically using in-house-built shell and Matlab scripts, implementing tools provided in the FMRIB’s Software Library toolbox (FSL, version 5.0.7, http://www.fmrib.ox.ac.uk).

The ischemic core on admission was defined applying an established and validated upper threshold of 620 × 10^−6^ mm^2^/s on apparent diffusion coefficient (ADC) maps [14]. Each individual automatically segmented ADC lesion was inspected thoroughly for accuracy and corrected manually, if necessary. During this procedure, the corresponding diffusion-weighted image (b = 1000 s/mm^2^) was taken into consideration. The segmented infarct core was used for volumetric assessment.

For automated quantitative and observer-independent assessment of collateral supply, T2*-weighted PWI time series were processed as described recently in detail [9, 10]. After motion correction of the T2*-weighted time series and calculation of maps representing voxels with a high coefficient of variance (CoV) in the signal-time curves after applying established thresholding, maps of the pial collateral vasculature were computed with a correction procedure. Finally, a collateral vessel index (CVI_PWI_) was calculated by dividing the volumetric abundance of collateral vessels along the lateral and cranial convexity of the affected by the unaffected side [10]. For the MR perfusion-based classification of favorable vs. poor to moderate collateral supply, a previously established CVI_PWI_ threshold of ≥ 0.96 [10] was applied as a cut-off value.

In order to assess the association of the CVI_PWI_ with a mismatch for endovascular thrombectomy (EVT) as defined in a randomized clinical trial yielding large effect sizes for EVT with regard to the clinical benefit in selected late window patients, the clinical/imaging mismatch criteria of the DAWN trial [15] were retrospectively applied to the patient cohort.

### Statistical Analysis

Categorical variables were described by count and percentage, and continuous and ordinal variables by median and interquartile range or mean ± SD. Inter-rater agreement for CTA-based ordinal collateral scores (forming the basis for dichotomized collateral scores) was determined using Cohen’s weighted kappa. The relationships between the various collateral measures (CTA-based collateral scores and MR-based CVI_PWI_) as well as between collateral supply and extension of the core infarct (ASPECTS on non-contrast CT and DWI, segmented ischemic core based on ADC) were assessed by correlation analyses (Spearman’s rank correlation). The Mann Whitney U‑test and the Kruskal Wallis H‑test were used to perform group comparisons. Stepwise binary logistic regression analyses with age, score on the National Institutes of Health Stroke Scale (NIHSS) on admission, EVT and successful reperfusion with TICI ≥ 2b as covariates were used to assess the association of CTA-based collateral scores and CVI_PWI_ with favorable early functional outcome (defined as a score of 0–2 on the modified Rankin Scale (mRS)). To avoid potential bias related to binarization, the full range of CTA-based collateral scores, respectively CVI_PWI_ values was used for these analyses. Statistical analyses were performed with JASP 0.18.3 (The University of Amsterdam, https://jasp-stats.org).

## Results

### Demographic and Clinical Baseline Characteristics

**Fig. 1:**
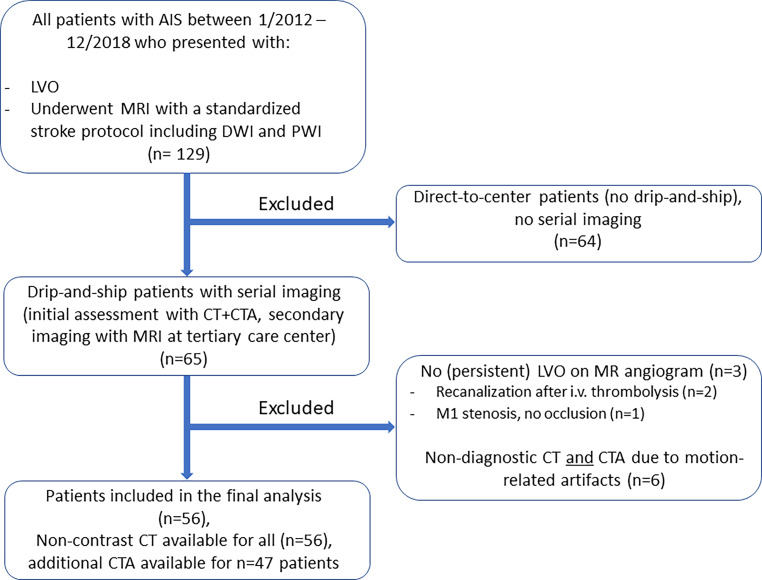
Population flowchart. Initially, *n* = 129 LVO patients who underwent MR imaging with a stroke protocol between 01/2012 and 12/2018 were identified from our database. After exclusion of *n* = 64 direct-to-center patients without serial imaging, an additional proportion (*n* = 9) of patients was excluded (*n* = 3 due to no (persistent) LVO at the time of MR imaging, *n* = 6 due to insufficient CT + CTA image quality). 56 patients were included in the final analysis. (*AIS* acute ischemic stroke, *LVO* large-vessel occlusion, *MRI* magnetic resonance imaging, *DWI* diffusion-weighted imaging, *PWI* perfusion-weighted imaging, *CT(A)* computed tomography (angiography))

A total of *n* = 129 LVO-AIS patients with MR imaging were identified in our database, of which *n* = 73 were excluded. Consequently, 56 patients (*n* = 31 (55.4%) female) were included in the final analysis, of which *n* = 47 had complete baseline CT imaging including single-phase CTA with an appropriate quality for systematic assessment of collateral supply. The population flowchart (Fig. 1) illustrates the process of patient selection for the final analysis. A detailed summary of demographic and clinical baseline characteristics of the final cohort is provided in Table 1.

**Table 1:** Demographic and clinical baseline characteristics of the entire patient cohort (*n* = 56)

*n*	56
Age [mean ± SD]	69.9 ± 14.21
Female sex [*n* (%)]	31 (55.4)
Pre-stroke mRS [median (IQR)]	0 (1–0)
Onset-to-CT imaging time [h] [median (IQR)]	2.3 (5.3–1.1)
*Stroke etiology [n (%)]*	
Cardioembolic	23 (41.1)
Large-artery disease	23 (41.1)
Other	4 (7.1)
unknown	6 (10.7)
Occlusion laterality, left [*n* (%)]	32 (57.1)
*Occlusion site [n (%)]*	
MCA M1	39 (69.6)
ICA + MCA M1	8 (14.3)
ICA	9 (16.1)
NIHSS admission [median (IQR)]	13.5 (18–8)
CT ASPECTS [median (IQR)]	8 (9–7)
i.v. thrombolysis [*n* (%)]	26 (46.4)
CT-to-MRI time [h] [median (IQR)]	2.5 (3.8–2.1)
Onset-to-MR imaging time [h] [median (IQR)]	5.2 (8.6–3.5)
DWI ASPECTS [median (IQR)]	7 (8–6)
Ischemic core volume [cm^3^] (mean ± SD)	29.9 ± 24.5
Clinical-Imaging mismatch [*n* (%)]	40 (17.4)
CVI_PWI_ [a.u.] (mean ± SD)	0.96 ± 0.242
EVT [*n* (%)]	40 (71.4)
Reperfusion with eTICI score 2b + [*n* (%)]	26 (46.4)
Onset-to-reperfusion time (ORT) [h] [median (IQR)]	7.8 (10.6–5.9)
NIHSS at 24 h [median (IQR)]	11 (18–5.5)
NIHSS discharge [median (IQR)]	6.5 (17.75–6.5)
mRS discharge [median (IQR)]	3 (5–2)

*SD* standard deviation, *mRS* modified Rankin Scale, *CT* computed tomography, *MCA* middle cerebral artery, *ICA* internal carotid artery, *NIHSS* National Institutes of Health Stroke Scale (NIHSS), *ASPECTS* Alberta Stroke Program Early CT Score, *h* hours, *MRI* magnetic resonance imaging, *IQR* interquartile range, *DWI* diffusion-weighted imaging, *cm*^*3*^ cubic centimeters, *CVI*_*PWI*_ Collateral vessel index in perfusion-weighted imaging, *EVT* endovascular thrombectomy, *eTICI score* expanded Thrombolysis in Cerebral Infarction score

### Associations Between CT-based Collateral Scores and MR-derived Quantitative Collateral Supply (CVI_PWI_)

**Fig. 2:**
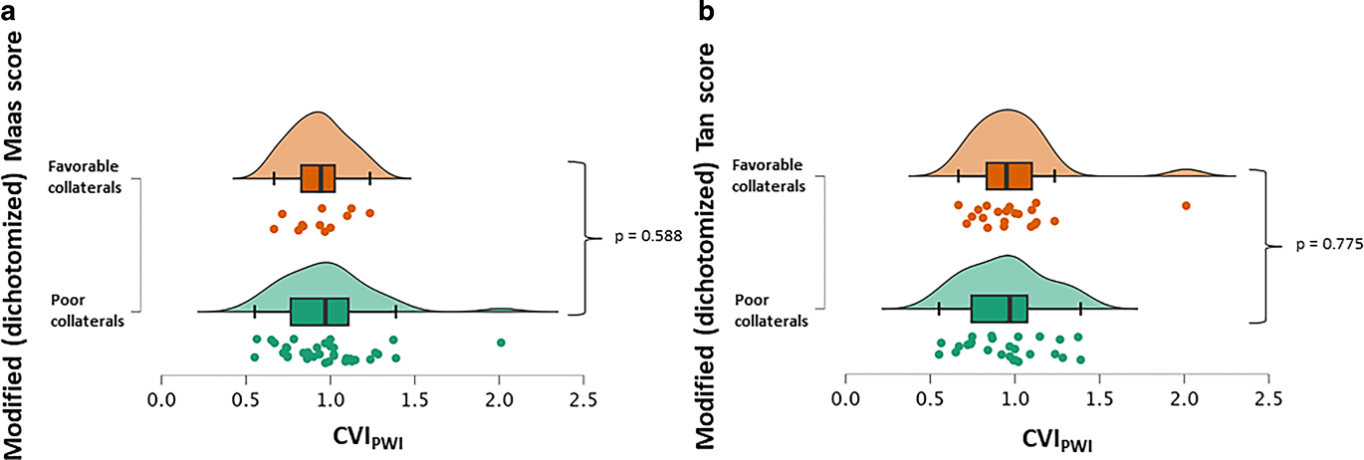
Raincloud plots for comparison of individual MR-based quantitative collateral supply (CVI_PWI_) after CT-based stratification of patients into groups of favorable and poor collateral supply with the modified (dichotomized) Maas (**a**) and Tan (**b**) score. (*CVI*_*PWI*_ Collateral vessel index in perfusion-weighted imaging)

Image evaluation for CTA-based collateral scores yielded a substantial, respectively high inter-rater agreement for the Tan (weighted κ = 0.791 (95% CI 0.642–0.940)) and Maas (weighted κ = 0.857 (95% CI 0.709–1.000)) collateral scores. No significant association was found between MR-based quantitative collateral supply (CVI_PWI_) and CT-based collateral scores (r = −0.00057, *p* = 0.502 and r = −0.124, *p* = 0.797). After dichotomization of the Tan and Maas score into favorable vs. poor collateral supply, there were no significant differences in MR-derived CVI_PWI_ between the respective groups (Fig. 2a, b). The proportion of patients with a favorable collateral profile according to the established CVI_PWI_ threshold did not differ between the groups of CT-based favorable vs. poor collateral supply (for Tan score: 47.6% vs. 53.8%, *p* = 0.671 and for Maas score: 41.7% vs. 54.3%, *p* = 0.450). In order to test for a potential association between prior i.v. thrombolysis and collateral status, the patient cohort was divided for group comparison. After stratification of the entire patient collective according to the administration of i.v. thrombolysis, there were no significant differences in terms of the MR-based quantitative collateral supply (CVI_PWI_ in patients with prior i.v. thrombolysis (*n* = 26) 0.999 ± 0.263 vs. 0.924 ± 0.223 in patients without prior i.v. thrombolysis (*p* = 0.487)) and CTA-based collateral status (*p* = 0.221 and *p* = 0.224) between the two groups.

### Relationship of Collateral Supply with Ischemic Core Volume and Clinical Stroke Severity

**Fig. 3:**
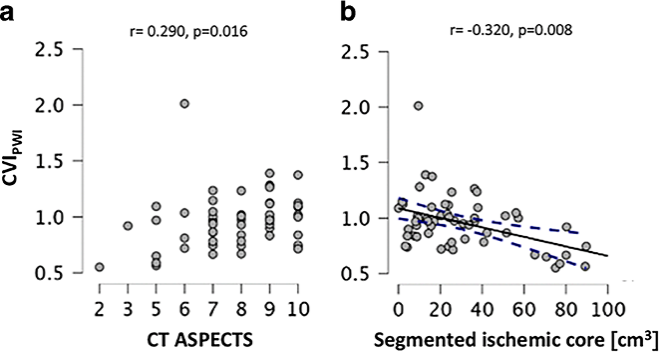
Scatterplots illustrating the relationship of MR-based quantitative pial collateral supply with CT ASPECTS (**a**) and automatically segmented ischemic core volume (**b**). (*CVI*_*PWI*_ Collateral vessel index in perfusion-weighted imaging, *CT* computed tomography, *ASPECTS* Alberta Stroke Program Early CT Score, *cm*^*3*^ cubic centimeters)

While MR-based CVI_PWI_ was significantly positively correlated with the ASPECTS on initial CT imaging (r = 0.290, *p* = 0.016, Fig. 3a), no significant relationship was found between CT-based collateral scores and CT ASPECTS (*p* = 0.152 and *p* = 0.211). None of the employed modalities for collateral assessment showed a significant correlation with DWI ASPECTS or with the decrease of ASPECTS over time from CT to MR imaging (all *p*-values > 0.27). Regarding the automatically segmented ischemic core volume on DWI, among the three collateral measures only CVI_PWI_ showed a significant negative relationship (r = −0.320, *p* = 0.008, Fig. 3b) with the core infarct.

**Fig. 4:**
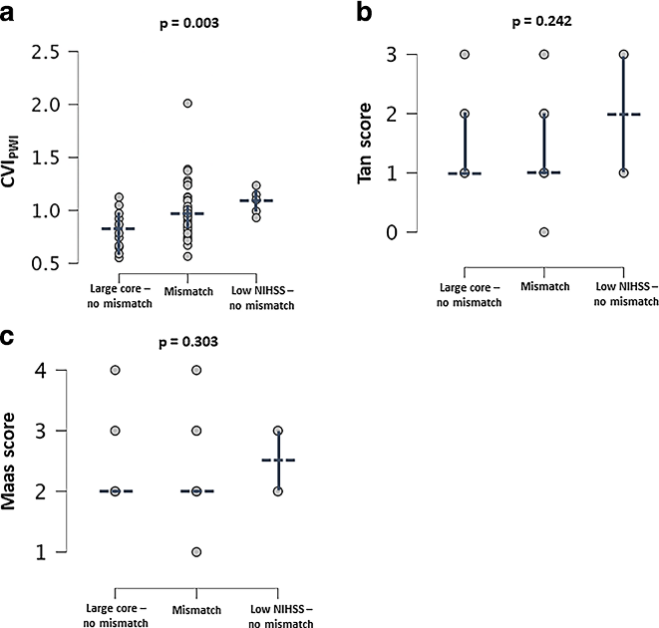
Scatterplots illustrating the relationships between the presence of a clinical-imaging mismatch and the degree of collateral supply in each of the employed imaging modalities for collateral grading. (**a** MR-based quantitative collateral supply (CVI_PWI_), **b** CT-based Tan score, **c** CT-based Maas score. In each panel, the median (dashed line) and interquartile range (solid line) for the respective collateral measures are shown in blue. *CVI*_*PWI*_ Collateral vessel index in perfusion-weighted imaging, *NIHSS* National Institutes of Health Stroke Scale)

More severe clinical deficit on the NIH stroke scale on admission was associated with lower CT ASPECTS and DWI ASPECTS (*p* < 0.001) and higher ischemic core volume (r = 0.322, *p* = 0.008). CVI_PWI_ showed a significant negative correlation with NIHSS on admission (r = −0.224, *p* = 0.049), while CT-based Tan and Maas score were not significantly correlated with clinical stroke severity (r = −0.148, *p* = 0.160 and r = −0.204, *p* = 0.084). Application of the DAWN criteria to the cohort resulted in three different groups: (1) patients not fulfilling clinical/imaging mismatch criteria due to a large infarct core on admission, (2) patients fulfilling clinical/imaging mismatch criteria and (3) patients not fulfilling clinical/imaging mismatch criteria due to a minor clinical deficit (low NIHSS). After subdivision of the entire patient cohort in these three groups, the MR-based CVI_PWI_ varied significantly across the groups (*p* = 0.003), while for the CTA-based Tan and Maas score no significant group differences were found (Fig. 4a–c).

### Association of Collateral Supply with Early Functional Outcome

In binary logistic regression for favorable early functional outcome (mRS 0–2 at discharge) with stepwise inclusion of the variables, in which age, NIHSS on admission, EVT and successful reperfusion with TICI ≥ 2b were entered as covariates, CVI_PWI_ remained independently significantly associated with favorable early functional outcome (Odds ratio (OR): 1.075, 95% CI 1.001–1.153, *p* = 0.046) together with initial stroke severity (NIHSS on admission) (OR 0.705, 95% CI 0.512–0.972, *p* = 0.033) (Table 2). In binary logistic regression with CT-based collateral scores for early functional outcome, using the same covariates, neither the Tan nor the Maas score was significantly associated with favorable functional outcome at discharge: OR 2.537, 95% CI 0.631–10.205, *p* = 0.190 for Tan and OR 5.817, 95% CI 0.475–71.247, *p* = 0.168 for Maas. When adding i.v. thrombolysis as a binary variable to the regression model, it was not significantly associated with favorable functional outcome (OR 0.698, 95% CI 0.035–14.080, *p* = 0.815). Furthermore, the associations of CVI_PWI_ (OR 1.071, 95% CI 1.001–1.136, *p* = 0.043) and baseline stroke severity (NIHSS) (OR 0.712, 95% CI 0.521–0.975, *p* = 0.034) with early functional outcome did not change relevantly after adding i.v. thrombolysis to the model. Among the patients with a good MR-based collateral profile (CVI_PWI_ ≥ 0.96, *n* = 31), the proportion of patients with favorable functional outcome was higher in patients with good collaterals who had received prior i.v. thrombolysis compared to patients with good collaterals who did not receive i.v. thrombolysis (53.8% vs. 33%), but the difference did not reach statistical significance (OR 2.333, 95% CI 0.539–10.109, *p* = 0.253). In order to evaluate the influence of time metrics on functional outcome, the relationship between the onset-to-reperfusion time (ORT), as an important measure incorporating various process times, and early functional outcome (ordinal score on the mRS at discharge) was investigated in the subgroup of patients, in which successful reperfusion (eTICI ≥ 2b) was achieved by EVT (*n* = 26). There was no statistically significant relationship between ORT and mRS at discharge (r = 0.086, *p* = 0.376). After stratification of the patients according to a favorable (*n* = 17) and unfavorable (*n* = 9) MR-based collateral profile (CVI_PWI_ ≥ 0.96), this relationship did not change relevantly and no significant correlation between ORT and mRS at discharge was detected (r = 0.037, *p* = 0.457 and r = −0.100, *p* = 0.608).

**Table 2:** Logistic regression analysis of CVI_PWI_ association with favorable early functional outcome (mRS 0–2 at discharge)

Variable	OR (95% CI)	*p*-value
CVI_PWI_	1.075 (1.001–1.153)	0.046
Age	0.945 (0.867–1.032)	0.207
NIHSS admission	0.705 (0.512–0.972)	0.033
EVT	4.821 (0.202–144.999)	0.331
Successful reperfusion (eTICI score ≥ 2b)	2.511 (0.158–39.849)	0.514

*CVI*_*PWI*_ Collateral vessel index in perfusion-weighted imaging, *NIHSS* National Institutes of Health Stroke Scale, *EVT* Endovascular thrombectomy, *eTICI* expanded Thrombolysis in Cerebral Infarction score

## Discussion

In this study, a recently established time-resolved and quantitative MRI-based approach for collateral mapping, which is independent from the application of grading systems [9, 10], was directly compared to commonly applied collateral scores [7, 11–13] based on conventional single-phase CTA in drip-and-ship patients who underwent stroke MR imaging after initial CT imaging for decision-making in terms of EVT.

CTA-based collateral scores did not show any significant relationship with initial stroke severity and infarct volume (CT and DWI ASPECTS as well as segmented ischemic core) and were not significantly associated with early functional outcome in multivariate analysis. Nearly half of the patients with poor collateral supply according to the modified Tan and Maas score exhibited a favorable collateral profile in time-resolved MR-based collateral mapping with a CVI_PWI_ value above the formerly established threshold for a good collateral supply [10]. Furthermore, no significant differences in quantitative collateral supply (CVI_PWI_) were found after subdivision of the patient cohort into groups with good and poor collateralization according to dichotomized CT-based collateral scores (Fig. 2a, b), suggesting that quantitative collateral profiles in time-resolved collateral imaging do not differ among the groups stratified according to CTA-based collateral status. The discrepancy between rater-dependent collateral status on CTA and time-resolved mapping of collateral supply cannot be interpreted without at least considering the time interval between CT and MR imaging in this study (median 2.5 h). The possibility of a change in collateral recruitment over time has been widely acknowledged and is a well-documented phenomenon, especially at later time points after stroke onset [16]. While some studies reported better collateral status at longer times from symptom onset [12, 17], suggesting a time-dependent recruitment of additional collateral vessels, others found less favorable collateral profiles at later time points of imaging after stroke onset [18], suggesting a deterioration of collateral blood flow over time. In addition, another study found no relationship at all between time from symptom onset to imaging and collateral status [19]. Unlike those studies, Uniken Venema et al. used an observer-independent quantitative method for direct collateral assessment in a large patient sample and did not find an association between time to imaging and collateral status, suggesting relative stability of collateral blood flow for several hours after stroke onset [20]. Given their results and the fact that CVI_PWI_ correlated significantly with the ASPECTS on initial non-contrast CT, it seems more likely that detected differences in the quality of collateral supply between CTA and MR-based CVI_PWI_ and in related clinical and outcome measures are primarily attributable to methodological differences and less likely to changes in collateral flow over time [20].

MR-based quantitative collateral mapping with CVI_PWI_ showed a significant positive correlation with ASPECTS on baseline CT imaging as well as with the ischemic core volume (Fig. 3a, b). The lack of a significant correlation with DWI ASPECTS is not entirely intuitive. Across the entire cohort, median ASPECTS changed rather slightly from CT to MR imaging (Table 1). Apart from changes of the infarct volume during the time interval between CT and MR imaging, especially different sensitivities of the imaging modalities to ischemic tissue changes [21] and the location-dependent but overall only moderate correlation of DWI ASPECTS with the ischemic core volume [22] have to be considered as reasons for the lack of a significant relationship between CVI_PWI_ and DWI ASPECTS. CTA-based collateral scores showed no significant relationship with ASPECTS and ischemic core volume, suggesting they do not accurately reflect the progression rate of the ischemic tissue damage in the acute phase, although a significant association between collaterals on CTA and final infarct volume has been shown in previous studies [7, 8, 23, 24]. In contrast to CTA-based collateral grading, CVI_PWI_ was a significantly associated with early functional outcome in multivariate analysis (Table 2) and showed significant variation across patient groups after stratification according to clinical/imaging mismatch criteria (Fig. 4a–c). These findings suggest that quantitative collateral assessment with CVI_PWI_ depicts leptomeningeal collateral supply, which is closely tied to initial stroke severity, progression of ischemic tissue damage and clinical response to reperfusion therapies. In contrast, commonly performed collateral grading with single-phase CTA and observer-dependent rating scales do not seem to appropriately reflect interindividual variation in collateral supply with clinical relevance. The discrepancies between CTA and CVI_PWI_ can be explained by rigidity of collateral grading systems, which do not capture smaller but still significant interindividual variations in collateral supply, and by important technical differences between the two methodologies, which are inherent to the scanning duration and the acquisition phase [8]. Those technical differences explain why MR-based quantitative collateral mapping is able to capture collateral flow via late-filling smaller leptomeningeal blood vessels, which is commonly missed by single-phase CTA as it mainly detects retrograde filling of larger arteries in the early phase after the contrast injection [7].

Patients with a favorable collateral profile in MR-based collateral mapping (CVI_PWI_ threshold) were nearly evenly distributed between patients with fair to good and poor collateral supply on CTA (∼50% in each group) and there were no significant group differences in CTA collateral scores after stratification based on clinical-imaging mismatch criteria (Fig. 3b, c). These results suggest that a relevant proportion of patients with an unfavorable collateral profile on baseline CTA may exhibit robust collateral blood flow on time-resolved collateral imaging and a considerable amount of salvageable tissue — even hours later. In patients with poor collateral supply on CTA, a favorable CVI_PWI_ at a later time point presumably reflects underestimation of collateralization by initial CTA (less likely augmentation of collateral recruitment over time), the opposite case most likely reflects early collateral failure over time. It is reasonable to assume that patients with a favorable collateral profile who underwent i.v. thrombolysis might have a higher likelihood of good outcome compared to patients with good collaterals without i.v. thrombolysis [25]. In the present study, we did not find any association between the collateral status and prior administration of i.v. thrombolysis. Our results point to a certain synergistic effect of leptomeningeal collaterals and i.v. thrombolysis with regard to achieving a favorable outcome, although the sample size might simply have been too small to detect this effect. However, in our data, we did not find any evidence that the positive effect of collaterals on the early functional outcome is mediated by i.v. thrombolysis. With regard to ORT as an important time metric, previous research demonstrated an association with functional outcome in patients with recanalization success [26]. The magnitude of the influence of ORT on functional outcome was found to be strongly dependent on the degree of collateral supply [26]. Presumably due to the small overall sample size, we could not replicate this finding in the subgroup of patients with successful reperfusion in our study — even after stratification of the patients according to collateral supply. The clinical implication of the presumable systematic underestimation of collateral supply on single-phase CTA is that patients with poor collateral flow on the initial CTA may in effect exhibit robust collateral profiles even in later time windows and consequently may benefit from EVT and achieve a favorable functional outcome. In line with previous research [2], the findings of our study highlight the relevance of the collateral status in later time windows after symptom onset or in cases with time delays to stroke treatment for clinical outcome prognostication and might justify additional pre-treatment stroke imaging to this end. In this case, time-resolved imaging methods may be preferable due to their ability to depict the actual magnitude of collateral flow more reliably. In late window patients or in cases with longer delays after initial CT-based evaluation, MRI with CVI_PWI_ may be a useful tool for additional imaging work-up in terms of mismatch assessment and outcome prediction. Previous research demonstrated that MRI-based CVI_PWI_ can be calculated from PWI time series on a standard computer with minimal postprocessing times [10]. Yet, despite those advantageous technical properties and the predictive value of CVI_PWI_ regarding imaging-based and clinical outcome parameters, its utility for the clinical acute setting depends on the software-based implementation of the postprocessing procedure on the scanner’s console. This would ensure the feasibility of MRI-based quantitative collateral assessment and its use for on-site therapeutic decision-making and outcome prognostication in acute ischemic stroke patients.

### Limitations

Some limitations of this study need to be addressed. One major limitation is the relatively small sample size, which might have an influence on our results and precludes the possibility of assessing the utility of collateral imaging modalities for selecting patients for EVT. In addition, multi-phase CTA, which was not used in this study, is superior to single-phase CTA with regard to the detection of collateral flow [27, 28] and may yield results which are more comparable to the results of quantitative collateral mapping with perfusion MRI. Yet, due to the lack of acquisition of multi-phase CTA for this study, we cannot comment on the utility of this important collateral imaging technique for functional outcome prediction in drip-and-ship patients in later time windows. Furthermore, due to the time interval (median: 2.5 h) between initial CT imaging and MR imaging with a standardized stroke protocol prior to EVT, the comparison between collateral profiles on baseline CTA and MRI is somewhat subject to uncertainty. Since recruitment of leptomeningeal collaterals is not inherently static over time, it cannot be excluded that CTA- and MRI-based collateral imaging results in this study are not directly comparable in every individual case, since due to additional collateral recruitment or collateral failure during the time interval between the imaging modalities, they might not reflect exactly the same collateral situation. Finally, the retrospective nature of this study might make our results prone to unknown sort of bias. Therefore, our findings should be interpreted with caution.

## Conclusions

Direct comparison revealed no significant association of collateral measures between conventional rater-dependent collateral scores based on single-phase CTA and quantitative and time-resolved MRI-based collateral mapping. Patients with poor collateral supply on CTA may exhibit a favorable collateral profile in time-resolved CVI_PWI_ associated with significant mismatch for thrombectomy and favorable clinical outcome, even hours after initial imaging, suggesting that single-phase CTA might underestimate leptomeningeal collateralization in a relevant proportion of patients. CVI_PWI_ represents a robust imaging marker of pial collateral supply and is associated with early functional outcome in LVO patients.
